# Salivary biomarkers for diagnosis of systemic diseases and malignant tumors. A systematic review

**DOI:** 10.4317/medoral.23355

**Published:** 2020-02-10

**Authors:** Marco Meleti, Diana Cassi, Paolo Vescovi, Giacomo Setti, Thelma A. Pertinhez, Margherita Eleonora Pezzi

**Affiliations:** 1 DDS, PhD. Researcher in Oral Medicine and Surgery. Centro Universitario di Odontoiatria, Department of Medicine and Surgery, University of Parma, Italy; 2 DDS, PhD, Spec. Orth.. Adjunct Professor. Department of Surgical, Medical, Dental and Morphological Science with interest in Transplant Oncological and Regenerative Medicine, University of Modena and Reggio Emilia, Italy; 3 DDS, MS, PhD. Spec. Or. Surg., Associate Professor of Oral Medicine and Surgery. Centro Universitario di Odontoiatria, Department of Medicine and Surgery, University of Parma, Italy; 4 DDS, PhD student. Centro Universitario di Odontoiatria, Department of Medicine and Surgery, University of Parma, Italy; 5 PhD. Associate Professor of Biochemistry. Department of Medicine and Surgery, University of Parma and Transfusion Medicine Unit, Azienda USL – IRCCS di Reggio Emilia, Italy; 6 DDS. Postgraduate student. Centro Universitario di Odontoiatria, Department of Medicine and Surgery, University of Parma, Italy

## Abstract

**Background:**

Saliva evaluation could be a possible alternative to blood and/or tissue analyses, for researching specific molecules associated to the presence of systemic diseases and malignancies. The present systematic review has been designed in order to answer to the question “are there significant associations between specific salivary biomarkers and diagnosis of systemic diseases or malignancies?”.

**Material and Methods:**

The Preferred Reporting Item for Systematic Reviews and Meta-analysis (PRISMA) statement was used to guide the review. The combinations of “saliva” and “systemic diseases” or “diagnosis” or “biomarkers” or “cancers” or “carcinoma” or “tumors”, were used to search Medline, Scopus and Web of Science databases. Endpoint of research has been set at May 2019. Studies were classified into 3 groups according to the type of disease investigated for diagnosis: 1) malignant tumors; 2) neurologic diseases and 3) inflammatory/metabolic/cardiovascular diseases. Assessment of quality has been assigned according to a series of questions proposed by the National Institute of Health. Level of evidence was assessed using the categories proposed in the Oxford Center for Evidence-Based medicine (CEMB) levels for diagnosis (2011).

**Results:**

Seventy-nine studies met the inclusion and exclusion criteria. Fifty-one (64%) investigated malignant tumors, 14 (17.5%) neurologic and 14 (18.5%) inflammatory/cardiovascular/metabolic diseases. Among studies investigating malignant tumors, 12 (23.5%) were scored as “good” and 11 of these reported statistically significant associations between salivary molecules and pathology. Two and 5 studies were found to have a good quality, among those evaluating the association between salivary biomarkers and neurologic and inflammatory/metabolic/cardiovascular diseases, respectively.

**Conclusions:**

The present systematic review confirms the existence of some “good” quality evidence to support the role of peculiar salivary biomarkers for diagnosis of systemic diseases (e.g. lung cancer and EGFR).

** Key words:**Salivary diagnostics, biomarkers, systemic diseases, malignant tumors, early diagnosis.

## Introduction

Currently, one of the most relevant targets of medicine and healthcare is early diagnosis. Detecting a disease at an early stage may improve the possibility of success of treatment, prevent complications and enhance prognosis and quality of life (1).

The concept of “point-of-care diagnosis” includes a field of investigation that explores technologies allowing patients and health providers to gain actionable medical information rapidly and conveniently (1).

The term “precision medicine” refers to the uses of molecular profiles, genomic, transcriptomic, proteomic and metabolomic, to adapt a personalized therapeutic strategy for peculiar patients: a. in the right moment, b. to determine the predisposition to diseases, and c. to provide timely and targeted prevention (2).

The combination of such profiles and the identification of biomarkers is leading to the development of new technologies, based on easy and non-invasive methods to collect diagnostic human specimens, possibly with a high specificity and sensitivity and customized on single patient.

In the last ten years, research has focused on the use of biomarkers in a previously poorly investigated human fluid: saliva.

Saliva is a fluid constantly produced by salivary glands and It has a complex molecular composition. Saliva is abundantly delivered in the oral cavity, its collection being simple and non-invasive. Moreover, transportation and storing are easy. For such reasons, saliva evaluation could be considered as a possible alternative to blood and/or tissue analyses, for researching specific molecules (DNA, RNA, proteins and metabolites) associated to the presence of systemic diseases and malignancies (3).

The present systematic review has been designed in order to answer to the question “are there significant associations between specific salivary biomarkers and diagnosis of systemic diseases or malignancies?”, formulated according to the “Patient-Intervention-Comparison-Outcome” (PICO) worksheet.

## Material and Methods

The Preferred Reporting Item for Systematic Reviews and Meta-analysis (PRISMA) statement was used to guide this systematic review (98).

- Search strategy

The combinations of “saliva” and “systemic diseases” or “diagnosis” or “biomarkers” or “cancers” or “carcinoma” or “tumors”, have been used for searching Medline, Scopus and Web of Science databases. Only English literature was searched. We considered articles published after 2000 (endpoint of research has been set at May 2019). Periodic screening of the databases was performed, between September 2017 and May 2019.

- Inclusion and exclusion criteria

Only papers reporting details on salivary sampling and biochemical analysis were included. Papers selected were primarily focused on the use of saliva for diagnostic purposes. We only included studies performed on humans, detailing the disease of patients and providing precise information on diagnosis. Studies reporting data on at least 5 patients were included.

Case reports, conference proceedings and personal communication were excluded.

Studies dealing with biomarkers evaluated for therapy, prognosis or staging of systemic diseases were not included.

We excluded researches specifically investigating salivary biomarkers in patients with systemic diseases with oral, oropharyngeal and esophageal involvement. Such a choice was taken in order to exclude presence of pathologies in the proximity of the site of saliva collection, thus avoiding confounding factors (e.g. contamination with peripheral molecules not originally presents in the salivary secrete).

Papers dealing with systemic microbial infections, hormones, drug dosage, were further excluded.

We excluded studies specifically reporting on biochemical methods, technological aspects, devices used or proposed for saliva evaluation or detection of specific molecules.

The criteria are summarized in Table 1.

**Table 1:**
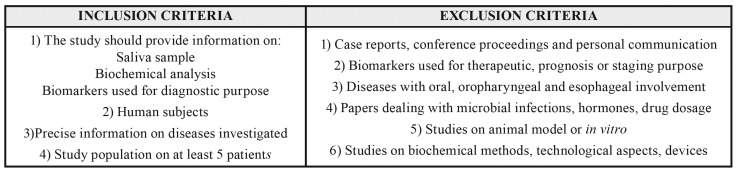
Inclusion and exclusion criteria.

- Data extraction

Titles and abstracts were screened by two independent investigators. Equivocal titles/abstracts were included for full-text evaluation.

Reviews of literature addressing the topic of salivary biomarkers and diagnosis of systemic diseases and malignant tumors were carefully read and all references were screened in order to include papers possibly not selected through the entry terms used within the databases. Other relevant literature was identified from the reference lists of the retrieved articles.

Information extracted from each study were summarized in an Excel Table and they included title, citation date (authors, publication year), pathology investigated, type of biomarkers, device used to analyze the sample, results and presence of statistically significant association.

- Data analysis

Studies were assessed for overlapping series of patients on the basis of the recruitment Centre and period. Wherever multiple studies reported the same set of data in fully detecTable overlapping series of patients, only the most recent or the most complete series was included in the review.

Studies were classified into 3 groups according to the type of disease investigated for diagnosis: 1) malignant tumors; 2) neurologic diseases and 3) inflammatory/metabolic/cardiovascular diseases.

- Quality assessment and critical appraisal

Assessment of quality has been assigned according to a series of questions proposed by the National Institute of Health (NIH) for each typology of study (controlled intervention studies, systematic reviews and meta-analysis, observational cohort and cross-sectional studies, case control studies, before-after studies with no control group, case series studies) (4).

Critical appraisal has been summarized through assignation of a value ranging from 0 to 100% to each of the study selected, based on the percentage of “yes” choices on the overall number of answers given. Furthermore, the number of patients enrolled in each study was taken into consideration.

Studies with a percentage of quality ranging from 80 to 100% were defined as “good”. Studies with a quality ranging between 50 and 80% were defined as “fair” and studies scoring less than 50% in quality were defined as “poor”.

Level of evidence was assessed using the categories proposed in the Oxford Center for Evidence-Based medicine (CEMB) levels for diagnosis [2011] (5).

Disagreement were resolved by discussion between the reviewers.

## Results

The systematic literature search provided 79 studies which met the inclusion and exclusion criteria.

Seventy-five papers were case-control studies and 4 case-series studies.

Fifty-one (64%) papers were focused on malignant tumors, 14 (17.5%) papers on neurologic diseases and 14 (18.5%) on inflammatory/cardiovascular/metabolic diseases (Table 2, Table 3, Table 4).

Molecules investigated were DNA, RNA, proteins, metabolites, microbiota and combination of these.

**Figure 1:**
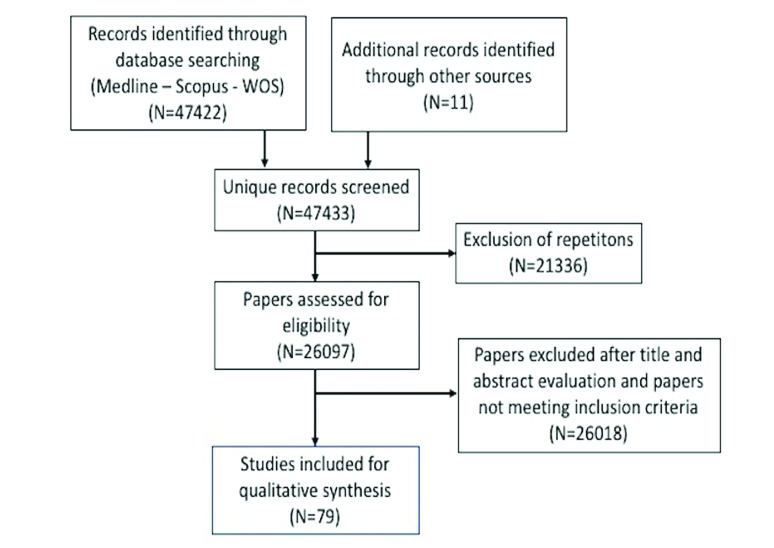
Search strategy.

Results of search strategy are summarized in Fig. 1.

**Table 2:**
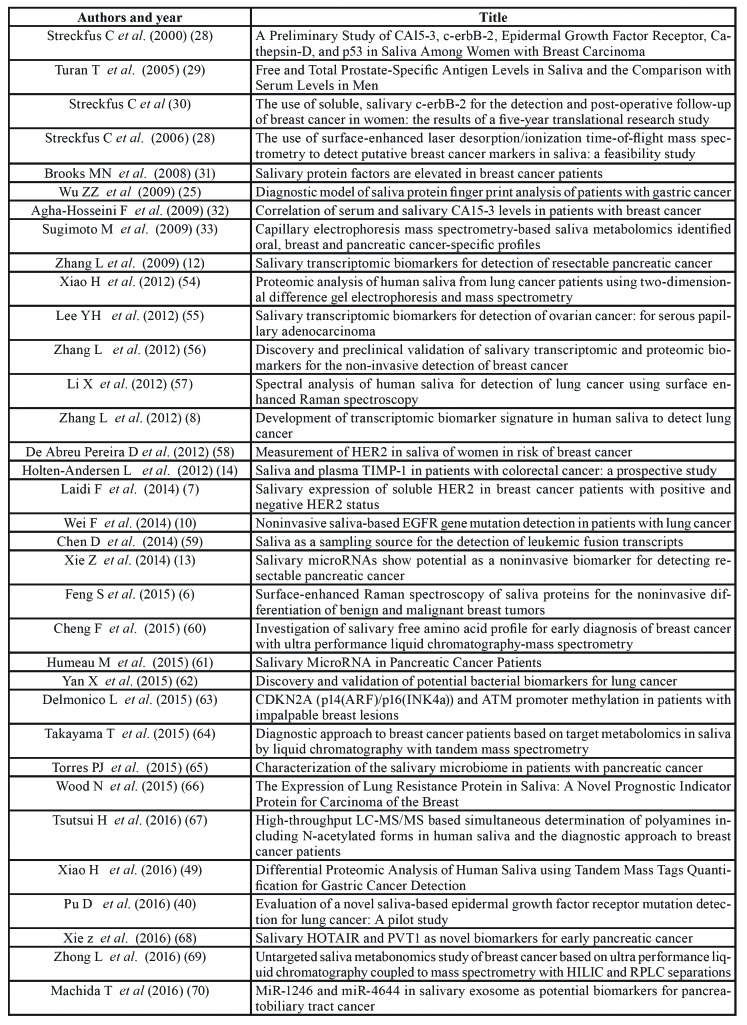
Papers on salivary biomarkers for diagnosis of malignant tumors.

**Table 2 cont.**
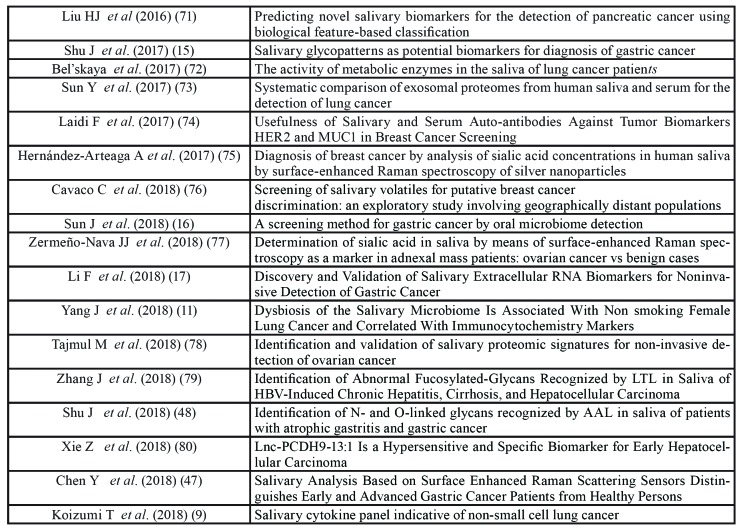
Papers on salivary biomarkers for diagnosis of malignant tumors.

**Table 3:**
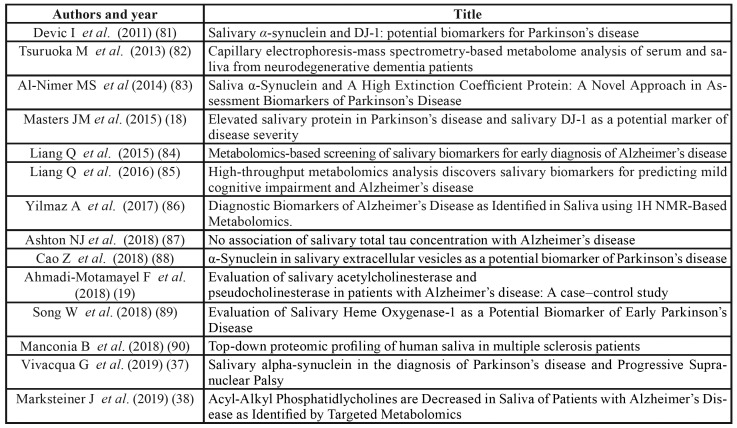
Papers on salivary biomarkers for diagnosis of neurologic diseases.

**Table 4:**
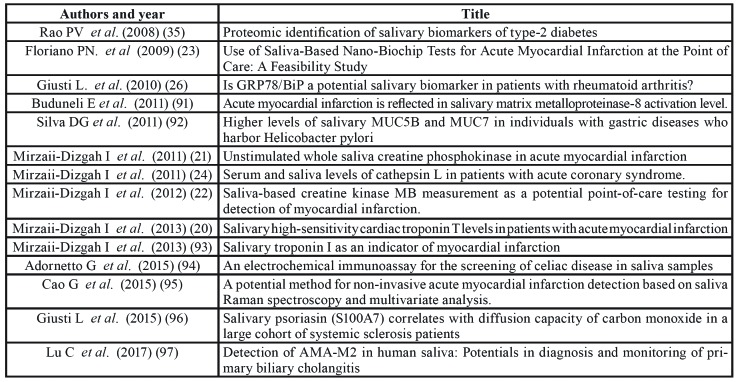
Papers on salivary biomarkers for diagnosis of inflammatory/cardiovascular/metabolic diseases.

- Critical appraisal of the selected papers

Following the NIH guidelines modified according to the methodology of the present systematic review, 19 (24%) studies were scored as “good”, 45 (57%) were “fair” and 15 (19%) had “poor” quality.

The most frequently encountered risk of bias (ROBs) were the absence of concurrent controls (71 papers), the lack of sample size justification (70 papers), the lack of randomization (70 papers) and absence of report of blinding exposure assessors (68 papers).

- Level of evidence

Application of the Oxford CEMB guidelines highlighted that all of the selected papers have a low level of evidence (4 on 5, the fifth level being the lowest), because of their case control or case series design.

- Malignant tumors

Breast cancer was the most investigated disease (18 papers), followed by lung cancer (10 papers), gastric cancer (7 papers) and pancreatic cancer (6 papers). Other cancers included leukemia, prostate, ovarian, colorectal, pancreatobiliary and hepatocellular cancer.

Molecules most frequently investigated were proteins and RNA.

Twelve studies (23.5%) were scored as “good” and 11 of these reported statistically significant associations between molecules searched and pathology (6-17).

The twelve studies are summarized in Table 5.

- Neurologic diseases

Fourteen papers were focused on neurologic diseases. Among these, 7 papers investigated Alzheimer’s disease, 6 investigated Parkinson’s diseases and one multiple sclerosis.

Two papers were scored as “good”. One study searched DJ-1 proteins in Parkinson’s patients (not statistically significant association) (18). The other one analyzed salivary metabolites in patient with Alzheimer’s disease and found a statistically significant association between disease and biomarkers (19).

Details on such papers are reported in Table 6.

- Inflammatory/cardiovascular/metabolic diseases

Fourteen papers were included in this category, the pathologies investigated being diabetes, myocardial infarction, rheumatoid arthritis, gastric diseases, celiac disease and coronary syndrome.

Myocardial infarction was the most frequently studied disease (7 papers, 50%).

“Good” quality articles were 5, four focused on myocardial infarction and one on coronary syndrome. All studies searched for proteins and 5 of these reported significant results. (20-24)

Papers are summarized in Table 7.

- Biochemical technologies

It goes beyond the aim of the present systematic review to critically discuss the biochemical mehods utilized in the studies included.

Almost all the studies on genetic molecules used Polymerase Chain Reaction (PCR) technique.

The studies on proteins used mainly Enzyme-Linked Immunosorbent Assays (ELISA) or Mass Spectrometry (MS). Few studies used Surface Enhanced Raman Scattering (SERS) and Luminex.

For searching metabolites, the preferred technique was MS.

**Table 5:**
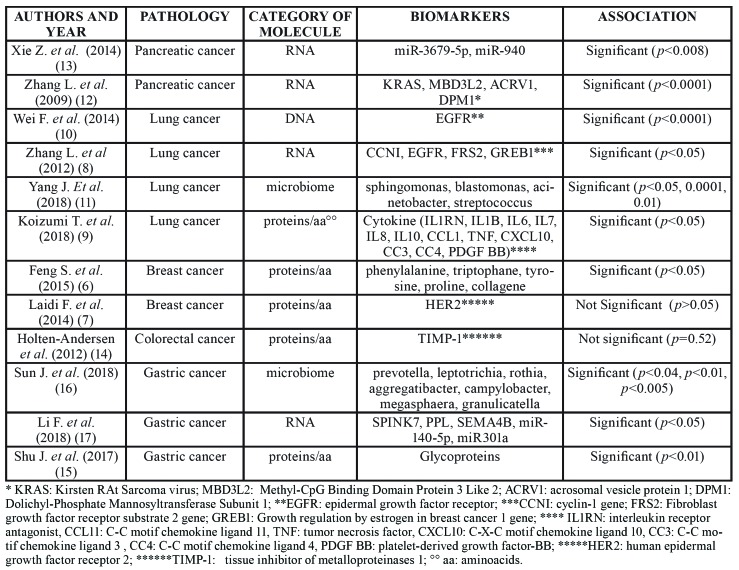
Good quality studies on salivary biomarkers for diagnosis of malignant tumors.

**Table 6:**
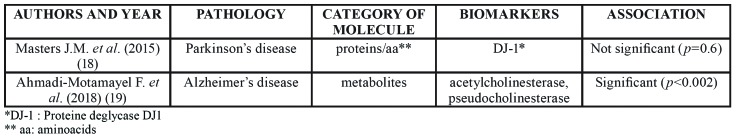
Good quality studies on salivary biomarkers for diagnosis of neurologic disease.

**Table 7:**
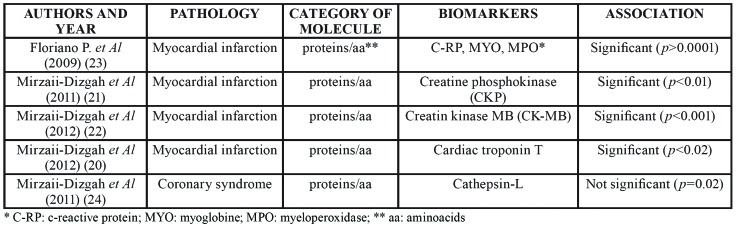
Good quality studies on salivary biomarkers for diagnosis of inflammatory/cardiovascular/metabolic diseases.

## Discussion

The present systematic review has highlighted an increased scientific interest toward the use of salivary biomarkers for diagnosis of systemic diseases and malignant tumors. Even limiting the field of interest only to diagnosis and applying strict inclusion and exclusion criteria, the number of papers appears considerable.

It is worthy to mention here that among the 79 papers included, only 12 studies were conducted before 2010, the rest being published after such year (12,23,25-34).

The most investigated salivary molecules are proteins (43 studies), followed by metabolites (15 studies) and RNA (12 studies). Surprisingly, the less studied salivary biomarkers are those based on DNA (3 studies) and microbiota (2 studies) analysis, despite their popularity for other aims (e.g ancestry investigations, biocompatibility for transplant, forensic analysis, dietary implications) (35,36).

In recent years, there has been a shifting of interest in the typology of molecules investigated. In fact, even if proteins remained predominant, there has been an increase of researches dealing with salivary metabolites (16 papers in 2018 and 2 papers in the first half of 2019) (37,38).

Most of the studies included in the present systematic review (69 out of 79 – 87%) showed statistically significant correlations between one or more biomarkers and specific pathologies. Such results would, in general, indicate the possibility to use peculiar salivary molecules for early diagnosis of diseases. However, critical appraisal and quality assessment highlighted that most of these studies did not satisfy a relevant percentage the items suggested by the NIH formats. As a matter of facts, only 19 studies received a score indicating “good” quality. Also, the level of evidence of all of the examined studies appears quite low.

One of the most frequently encountered ROBs was the lack of sample size justification. Calculation of a sample size is a fundamental step for creating reliable researches. Groups of patients too small have little chance of meeting the study objectives (39). Therefore, particularly for studies on salivary biomarkers it seems very important to report the justification of the population size. On the other hand, it should be taken into account that several studies included in the present review were pilot studies (27,40). For such a typology of research, it is usually difficult to provide a statistical reliable sample size justification, on the basis of the absence of background data in the literature (41). It is opinion of the Authors of the present systematic review that, the described ROB may largely depend on the fact that many of the studies took into consideration many variables at the same time (e.g. panel or combinations of very different biomolecules, biomarkers evaluated for the first time in saliva, patients with diseases at different stages), thus making more or less impossible to calculate a reliable sample size.

The second most frequently encountered ROBs involved the absence of blinding of exposure assessor. Blinding is important to remove bias that could influence the way the data is processed. The two major biases that can be controlled using blinding are the performance bias (differences that occur due to knowledge of intervention allocation, in either the researcher or the participant that cause differences in the care received) and the ascertainment bias (when data for a study or analysis is collected, surveyed, screened, or recorded, such that some members of the intended population are less likely to be included than others) (42). In the studies evaluated, the ROBs “absence of blindness” was induced by the fact that it was not specified if the biochemical analyst was or was not unaware of the provenance of the specimen (e.g. case or control group).

The most investigated disease in the present review was breast cancer (18 out of 79 studies), the most common malignant tumor among women (25% off all females tumors), with approximately 1.7 million new cases diagnosed every year (43). Fourteen studies (78%) reported statistically significant association between the presence of breast tumor and finding of one or more markers in patients saliva. Molecules such C-erbB2, CA 15-3, Cathepsin D, sialic acid and P53, EGF, VEGF and the CEA, seem to be promising salivary markers possibly very useful either for diagnosis of breast carcinoma and for follow-up of patients after treatment. According to the quality assessment tool adopted here, only one study dealing with breast cancer and salivary biomarkers obtained a “good” quality score, its results being apparently very robust (6). However, such a study investigated a panel of proteins, detected trough the SERS technology, which are not yet completely characterized and identified (6). Therefore, the utility of these proteins is currently somewhat questionable and their role should be confirmed in further studies.

The second most studied malignant tumor, according to the present systematic review is lung carcinoma (10 studies). Lung cancer is the most common cancer in humans (11.6% of all malignant tumours) and the leading cause of death for malignancy (18.4%) (44). All of the researches on patients with lung carcinomas provided statistically significant results to the association between salivary molecules and the pulmonary malignancy. Specifically, molecules identified included a mutation of the EGFR gene, 5 mRNA (CCNI, EGFR, FGF 19, FRS 2, GREB1), several bacteria (e.g. Sphingomonas, Blastomonas, Acinetobacter, *Streptococcus*) and proteins such as the calprotectin, alkaline phosphatase, cytokines, AZGP1 and haptoglobine (HP). All the reported molecules showed a good statistical association with diagnosis of lung carcinomas at different stages of developement. The “good” quality studies on salivary biomarkers and lung cancer are those demonstrating an association with EGFR, the 5 mRNA, microbiota and cytokines (8-11). According to the results of the present review they can be considered already reliable markers.

Data on association between salivary biomarkers and diagnosis of malignant tumors are available also for gastric and pancreatic cancer. Gastric cancer affects approximately one million individuals per year worldwide, having a mortality rate of approximately (1.033.701 new cases in 2018 and 782.685 death in 2018, in the world) (44). It is often detected late because up to 80 % of patients are asymptomatic during the early phases of disease (45). Similarly, pancreatic carcinoma is insidious, very aggressive and in most cases diagnosed at a very late stage, being associated to a very poor prognosis (46).

With regard to gastric cancer 7 studies were included in the present review (15-17,25,47-49). All of these reported statistically significant results. The “good” quality studies were three and they were focused on salivary bacteria (*Prevotella*, *Leptotrichia*, *Rothia*, *Aggregatibacter*, *Campylobacter*, *Megasphaera*, *Granulicatella*), RNA (SPINK7, PPL, SEMA4B, miR140-5p, miR301a) and some lectins.

All of the studies on pancreatic cancer demonstrated a significant association between the salivary molecules and the disease. Two of these were also scored as “good” after quality assessment. Molecules reported in such analysis were derived from transcriptomics (4mRNA (KRAS, MBD3L2, ACRV1, DPM1), and 2 miRNA (miR-3679-5p and miR-940)).

It is worthy to mention here that both for gastric and pancreatic cancer, the possibility of early diagnosis through salivary diagnostics, not based on the subjective radiographical images interpretation, could potentially contribute to prevent most of the deaths related to such cancers (3).

Particularly in the field of oncology, an easy and non-invasive method based on salivary biomarkers, may hypothetically constantly monitor and screen saliva, thus detecting very recurrences very (50).

Studies on neurological disorders are focused on Parkinson’s and Alzheimer's diseases. It is interesting to highlight that the use of salivary biomarkers in patients with neurological pathologies has gained a great interest in the last couple of years (8 out 14 articles published in 2017-18). Such an increasing of interest might be explained taking into consideration that the diagnosis of these diseases are essentially clinical. The identification of objective features (including biomolecules within body fluid) seems therefore of paramount importance for diagnosis, monitoring of disease progression and management as well as the development of novel therapeutic interventions (51).

Among studies on inflammatory, cardiovascular and metabolic diseases those reporting significant results are focused on myocardial infarction and rheumatoid arthritis (20-24,26).

Research on myocardial infarction searched C-reactive protein, myoglobin, myeloperoxidase, creatine phosphokinase, creatin kinase MB, cardiac troponin T and cathepsin-L, all reporting statistically significant result. In one of these studies the use a saliva-based nanochip was proposed (23). Such a new technology, based on nanotechnologies and innovative materials, is apparently very promising, deserving further researches.

Rheumatoid arthritis is sometime difficult to diagnose, especially in early stages, because of the variability of the symptoms and the absence of specific markers (52). Early diagnosis of the disease usually improves the success of treatment and could possibly reduce the quantity of drugs administered. In the study on included in the present review, Authors identified particularly one salivary protein (GRP78/BiP) which was significantly associated to the disease (*p*<0.001, 83.3% sensitivity and 95% specificity) (26).

A limit of the present systematic review is the lack of a quantitative analysis. The heterogeneity of diseases evaluated, their stage at diagnosis, the extremely wide range of molecules investigated as well as the differences in procedures for saliva collection, handling, storing and processing, makes it impossible to draw reliable pooled results or to perform a meta-analysis. On the other hand, the results of the present qualitative analysis can provide useful information in the field of salivary diagnostics. Particularly, the findings reported here can be the background for further studies which possibly might take into account the ROBs highlighted in the qualitative analysis of papers. Future studies might be useful to confirm and improve the potentiality of salivary analysis techniques (in terms of sensitivity and specificity) as well as to develop new, smaller, patient-friendly devices possibly with affordable costs.

Diffusion and ready availability of a panel of sensors for detecting salivary biomarkers associated to systemic diseases could have a strong impact on public healthcare and economy. The use of saliva for analysis might replace the use of blood, with a possible economic impact based on the easier and non-invasive method for collection (50). Such a perspective, may well lead to a higher commercial availability of screening assays (53) and possibly bring to the development of analytic tools directly administered in the dental or general physician office.

The use of salivary biomarkers for diagnosis of systemic diseases (“salivary diagnostics”) is gaining increasing interest.

The present systematic review confirms the existence of some “good” quality evidence to support the role of peculiar salivary biomarkers for diagnosis of systemic diseases (e.g. lung cancer and EGFR). However, it seems necessary to encourage further researches for improving the sensitivity and specificity of salivary diagnostics analysis.

The perspective of realizing a reliable “lab-on-a-chip” for diagnosis and follow-up of systemic diseases and/or malignant tumors through saliva evaluation seems an attainable target of modern medicine.
